# IL-6-Dependent PGE2 Secretion by Mesenchymal Stem Cells Inhibits Local Inflammation in Experimental Arthritis

**DOI:** 10.1371/journal.pone.0014247

**Published:** 2010-12-07

**Authors:** Carine Bouffi, Claire Bony, Gabriel Courties, Christian Jorgensen, Danièle Noël

**Affiliations:** 1 Inserm, Unité 844, Montpellier, France; 2 Université Montpellier 1, Montpellier, France; 3 Service d'Immuno-Rhumatologie Thérapeutique, Hôpital Lapeyronie, Montpellier, France; Ohio State University, United States of America

## Abstract

**Background:**

Based on their capacity to suppress immune responses, multipotent mesenchymal stromal cells (MSC) are intensively studied for various clinical applications. Although it has been shown i*n vitro* that the immunomodulatory effect of MSCs mainly occurs through the secretion of soluble mediators, the mechanism is still not completely understood. The aim of the present study was to better understand the mechanisms underlying the suppressive effect of MSCs *in vivo*, using cells isolated from mice deficient in the production of inducible nitric oxide synthase (iNOS) or interleukin (IL)-6 in the murine model of collagen-induced arthritis.

**Principal Findings:**

In the present study, we show that primary murine MSCs from various strains of mice or isolated from mice deficient for iNOS or IL-6 exhibit different immunosuppressive potential. The immunomodulatory function of MSCs was mainly attributed to IL-6-dependent secretion of prostaglandin E2 (PGE2) with a minor role for NO. To address the role of these molecules *in vivo*, we used the collagen-induced arthritis as an experimental model of immune-mediated disorder. MSCs effectively inhibited collagen-induced inflammation during a narrow therapeutic window. In contrast to wild type MSCs, IL-6-deficient MSCs and to a lesser extent iNOS-deficient MSCs were not able to reduce the clinical signs of arthritis. Finally, we show that, independently of NO or IL-6 secretion or Treg cell induction, MSCs modulate the host response by inducing a switch to a Th2 immune response.

**Significance:**

Our data indicate that MSCs mediate their immunosuppressive effect via two modes of action: locally, they reduce inflammation through the secretion of anti-proliferative mediators, such as NO and mainly PGE2, and systemically they switch the host response from a Th1/Th17 towards a Th2 immune profile.

## Introduction

Multipotent mesenchymal stromal cells or mesenchymal stem cells (MSC) are adult progenitor cells essentially isolated from bone marrow or adipose tissue that can be rapidly expanded *ex vivo* in large numbers. These cells are currently under investigation for tissue engineering applications, in particular for bone and cartilage repair, due to their potential to differentiate into the chondrocyte, osteoblast or adipocyte lineages [1]. Besides their differentiation properties, they may have a therapeutic value in other clinical applications based on their capacity to limit scar formation through anti-fibrotic properties, to prevent apoptosis, to stimulate the regeneration of endogenous cells and to suppress the host immune response (for review, see [2]). These immunosuppressive effects have been shown to occur mainly through the secretion of soluble factors. Among the possible mediators identified, indoleamine 2,3-dioxygenase (IDO) [3], inducible nitric oxide synthase (iNOS) [4]–[5], heme oxygenase (HO)-1 [6] as well as the secretion of human leukocyte antigen (HLA)-G [7], transforming growth factor (TGF)-β [8], interleukin (IL)-6 [9] and prostaglandin E2 (PGE2) [10] have been postulated to play a role in this process. Depending on the species, immunosuppression mechanisms displayed by MSCs may however differ. For instance, it has been shown that murine MSCs do not possess IDO activity, whereas human MSCs are devoid of iNOS (for review, see [11]). These mechanisms result in the inhibition of the proliferation of CD4^+^ and CD8^+^ T cells, B lymphocytes, NK cells that has been mainly shown *in vitro* but also *in vivo* in a number of experimental models reviewed in [12].

The therapeutic efficacy of MSCs has been evaluated in experimental autoimmune models, as well as in humans, to prevent acute graft versus host disease (GVHD) [13]. Zappia and collaborators were among the first to report the therapeutic efficacy of MSCs in the experimental autoimmune encephalomyelitis (EAE) [14]. In this murine model of multiple sclerosis, the administration of MSCs was found to decrease the clinical signs associated with demyelination when injected before or at disease onset. However, no therapeutic effect was observed when the injection occurred after disease stabilization. Similar results were observed in a model of autoimmune diabetes, where MSC injection promoted repair of pancreatic islets and renal glomeruli, as well as mesangial thickening and reduction in macrophage infiltration resulting in the prevention of pancreatic injury [15].

In collagen-induced arthritis (CIA), an experimental model of rheumatoid arthritis (RA), conflicting results on the role of MSCs have been reported. The first study on the use of MSCs in CIA showed that allogeneic C3H10T1/2 cells did not exert a beneficial effect on disease progression [16]. More recently, it has been demonstrated that systemic injection of MSCs, engineered to constitutively produce IL-10, after the recall of immunization significantly reduced the arthritic symptoms, in contrast to the lack of efficacy of wild type MSCs [17]. Since, it has been reported that a single injection of primary MSCs prevented the development of severe arthritis which was associated with a decreased level of pro-inflammatory cytokines in the sera of MSC-injected mice and an increased frequency of peripheral regulatory T (Treg) cells [18]. Similar results have been obtained *in vitro* and *in vivo* with human adipose-derived stem cells (ADSC) that were shown to suppress T cell responses through the generation and activation of antigen-specific Treg cells [19], [ 20].

The aim of our study was to elucidate the *in vivo* mechanisms of MSC-mediated immune suppression, in particular the role of IL-6, PGE2 and NO, the function of which is poorly investigated *in vivo*, and to evaluate their impact on T cell subpopulation in a model of inflammatory autoimmune disease. Contrary to numerous previous reports, we used highly characterized MSCs, either syngeneic or allogeneic and, MSCs deficient in the production of IL-6 or NO that are proposed to play an important role in their immunomodulatory function.

## Results

### Primary MSCs from different mouse strains are phenotypically and functionally different

**Figure 1:**
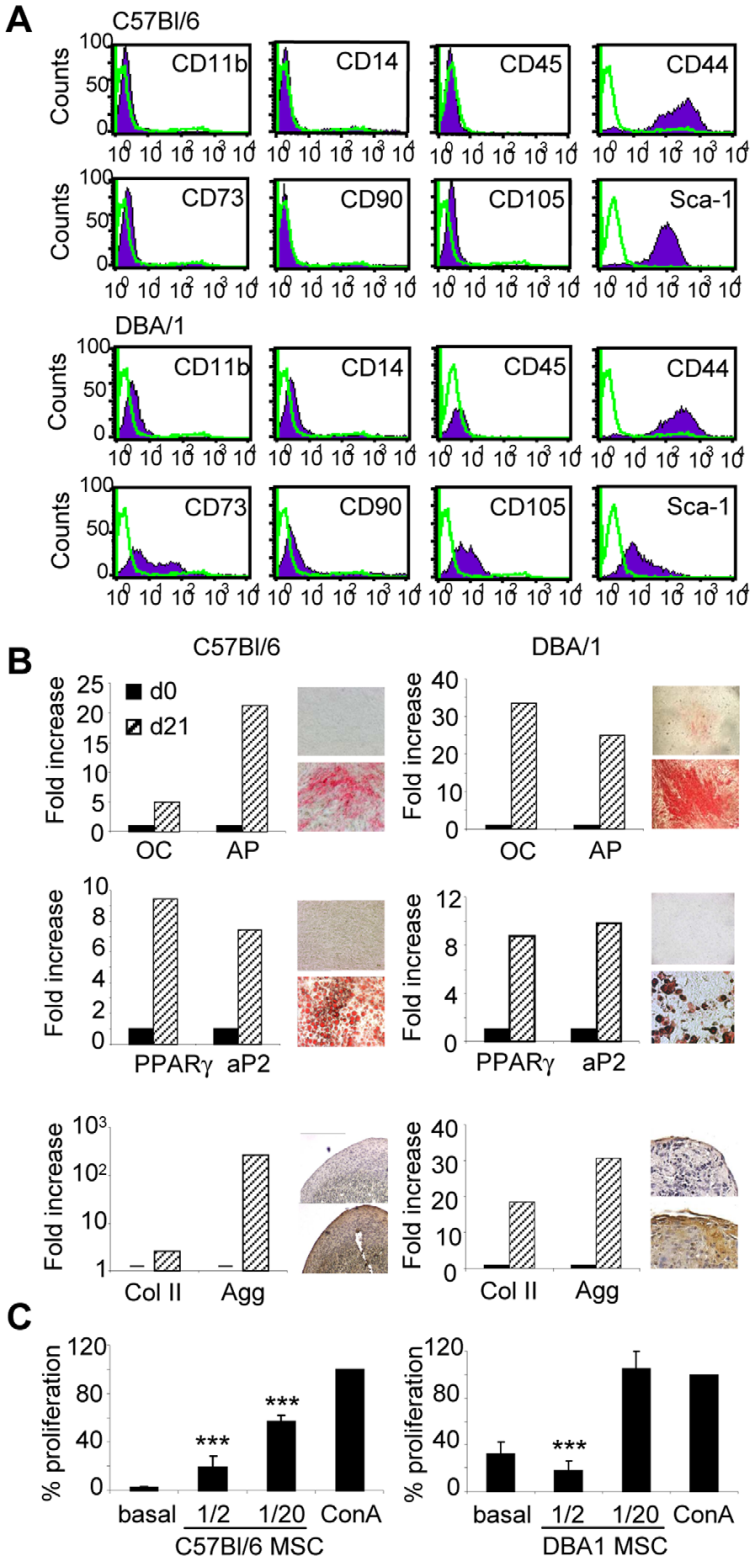
Phenotypic and functional characterization of primary murine MSCs. A) Immunophenotype of bone marrow-derived MSCs isolated from C57BL/6 and DBA/1 mice. Cells were stained with antibodies against the indicated antigens and analysed by flow cytometry. Representative histograms are shown in black and the respective isotype controls are shown as grey line and are representative of three experiments. B) Differentiation potential of murine MSCs. The differentiation in the 3 lineages is shown by up-regulation of specific markers by RT-qPCR and specific staining. Osteogenesis is characterized by the expression of osteocalcin (OC), alkaline phosphatase (AP) and Alizarin Red S positive staining in differentiation medium (lower panel) versus proliferative medium (upper panel). Adipogenesis is characterized by the expression of peroxysome proliferator-activated receptor (PPAR)-γ; fatty acid binding protein 4 (aP2) and Oil Red O positive staining in differentiation medium (lower panel) versus proliferative medium (upper panel). Chondrogenesis is characterized by the expression of collagen II (Col II) and aggrecan (Agg) and anti-aggrecan positive staining on pellet sections (lower panel) versus unstained control section (upper panel). Results are representative of three independent experiments. C) Immunosuppressive potential of murine C57BL/6 (left panel) and DBA/1 (right panel) MSCs. Allogeneic splenocytes were incubated for three days with ConA without or with various ratios of MSCs. Results are expressed as the percentage of ConA-induced proliferation which was assigned the value of 100%± SEM (n = 3; ***: p<0.005).

Because most of the *in vivo* studies reported so far relied on the use of poorly characterized murine MSCs, we decided to use a population of BM-derived cells satisfying the criteria used for MSCs. BM-derived cells, obtained from C57Bl6 or DBA1 mice, were first selected by plastic adherence. A long process of culture expansion was required to obtain a homogeneous cell population with a spindle-shaped fibroblastic morphology and that lacked hematopoietic markers. At this stage of culture, typically after passage 6, both cell types derived from C57Bl6 and DBA1 mice, named thereafter B6 and D1 MSCs respectively, were negative for CD11b, CD14 and CD45 and possessed cell surface molecules selectively expressed on MSCs including CD44 and Sca-1 (Fig. 1A). CD73 and CD105 were detected solely on D1 cells, whereas CD90 was absent on both B6 and D1 cells. The MSC nature of these cells was confirmed by their capacity to differentiate into three lineages. Expression of lineage-specific markers and components of extracellular matrix was respectively quantified by RT-qPCR and immunohistochemistry. Both D1 and B6 cells exhibited a similar potential to give rise to osteoblasts, as shown by an increase in osteocalcine, alkaline phosphatase and mineralization of the extracellular matrix; adipocytes, as shown by the expression of peroxysome proliferator-activated receptor γ, fatty acid binding protein 4 and formation of lipid droplets as well as chondrocytes, indicated by an enhanced expression of collagen II and aggrecan, both at transcriptional and protein level (Fig. 1B). As expected, these MSC populations exerted an inhibitory effect on the proliferation of allogeneic splenocytes stimulated by concanavalin A (Fig. 1C) which was dose-dependent, showing approximately a 80% to 100% inhibition of splenocyte proliferation at the highest ratio tested of one MSC for two splenocytes. The inhibitory effect was still detectable at the 1∶20 ratio for B6 MSCs whereas it was abolished for D1 MSCs at this concentration, suggesting a different suppressive potential depending on the mouse strain.

### MSCs improve the clinical signs of arthritis during a narrow therapeutic window

**Figure 2:**
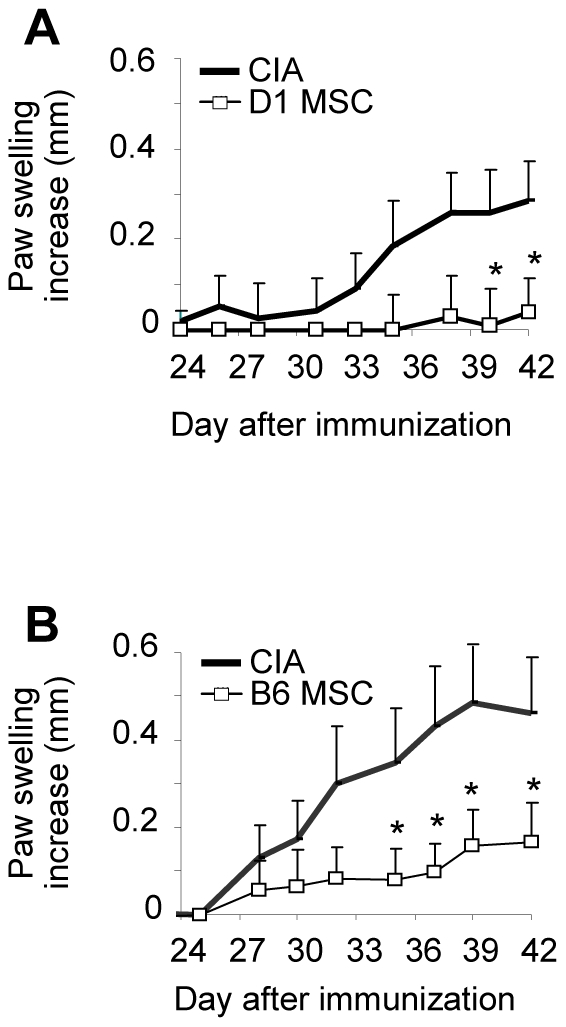
Immunosuppressive effect of syngeneic and allogeneic primary murine MSCs in the CIA model. A) Evaluation of the severity of arthritis by measurement of the increase of the paw swelling of CIA control mice or mice injected with syngeneic D1 MSCs on day 18 and day 24. Results are representative of two independent experiments. B) Evaluation of the severity of arthritis following injection of allogeneic B6 MSCs at d18 and d24. Results are representative of three independent experiments (*: p<0.05).

To determine *in vivo* the role of MSCs, we relied on the CIA model of autoimmune rheumatoid arthritis. In this model, conflicting results have been published on the therapeutic effect of MSCs. Some of them may be explained by the use of MHC-matched and -mismatched MSCs or the time of injection. First, we investigated the effect of D1 or B6 MSCs when injected intravenously in the DBA1 mice on day 18 and 24, 3 days before and after the boost with collagen II. Injection of either allogeneic B6 or syngeneic D1 MSCs resulted in a statistically significant inhibition of the paw swelling (Fig. 2A, 2B). In these experiments, both the clinical scores (mean score of 2.2±1 with B6 MSCs versus 4.2±1.4 in control and 3.5±0.9 with D1 MSCs versus 7.6±1.7 in control) and the delay of disease onset (day 32.5±1.7 with B6 MSCs versus 28.2±0.9 in control and day 37.33±0.7 with D1 MSCs versus 29.5±0.4 in control) were also statistically different. Hence, both syngeneic and allogeneic MSCs were effective in reducing the clinical signs of arthritis.

**Figure 3:**
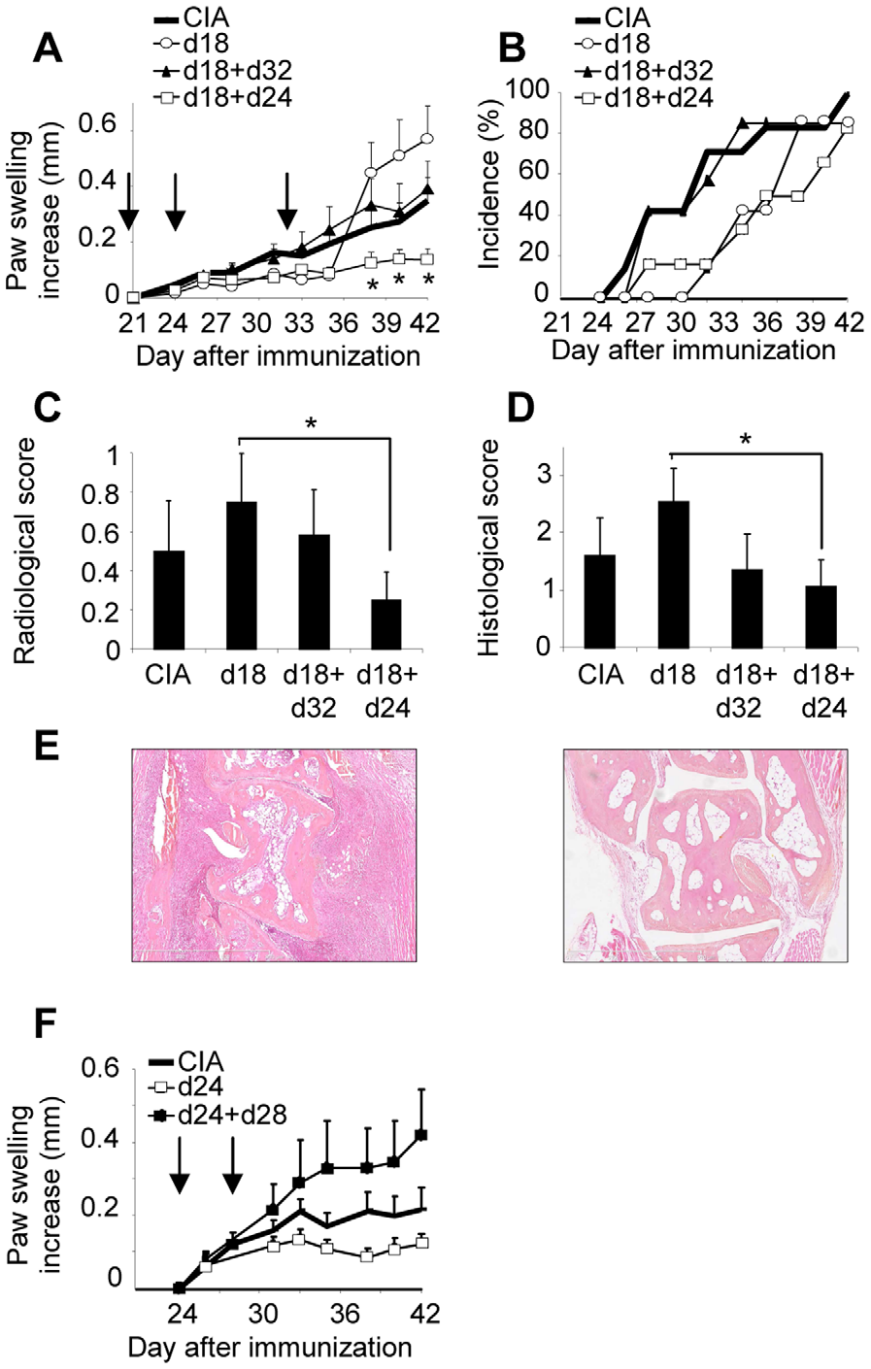
Immunosuppression is dependent of the injection time of primary MSCs in experimental arthritis. A) Evaluation of the increase of the paw swelling of CIA control mice or mice injected with syngeneic D1 MSCs at various time points. Results are expressed as mean increase ± SEM and are representative of three independent experiments. B) Incidence of arthritis evaluating the percentage of mice with a clinical score >1 (see experimental procedures). C) Radiological score evaluated after X-ray examination on hind paws from mice after euthanasia as assessed in experimental procedures. Results are expressed as the mean ± SEM. D) Histological score evaluated on paraffin sections of hind paws stained by hematoxylin-eosin as assessed in experimental procedures. Results are expressed as the mean ± SEM. E) Representative photomicrographs of joints from CIA control mice (left), or mice injected with MSCs on d18 and d24 (right). Magnification ×5. F) Evaluation of the paw swelling in CIA following injection of D1 MSCs after disease onset. Results are representative of two independent experiments (Statistical analysis showed not significance between groups).

Next, we tested the effect of single or repeated injections of syngeneic D1 MSCs performed at different time points. A single MSC injection on day 18 delayed the arthritis onset (34.7±1.4 versus 30±3.3 in CIA control mice), followed by a dramatic increase of the paw swelling by day 38 (Fig. 3A). Injection of MSCs on days 18 and 24 resulted in a significant reduction of the paw swelling. However, no effect on the paw swelling was observed, when the cells were injected on days 18 and 32. We therefore confirmed that injection of MSCs apart from the boost was beneficial for the treated mice. Repeated injection on days 18 and 24 was associated with a lower incidence (Fig. 3B), a delayed onset (35.7±2.6 for d18- and d24-injected mice versus 30±3.3 for CIA control mice) and a significant reduction in the average of the maximal paw swelling observed in each mouse during the course of the disease (2.1±0.06 versus 2.6±0.15, respectively). The anti-arthritic effect was further confirmed at the radiological and histological level (Fig. 3C-D). In addition, a reduced infiltration of immune cells into the joints was observed in mice treated with MSCs on day 18 and 24, as shown by histological analysis (Fig. 3E). We then evaluated whether MSCs were still effective when administered after the boost. A single or repeated administration of MSCs, either on day 24 or days 24 and 28 after disease onset, did not yield significant reduction of the paw swelling. Compared to control mice, repeated administration of MSCs even tended to exacerbate arthritis (Fig. 3F). Together, our results show that, independently of MHC haplotype, MSCs display a therapeutic effect when administered during a narrow therapeutic window.

### IL-6 is the central player in MSC immunomodulatory properties

**Figure 4:**
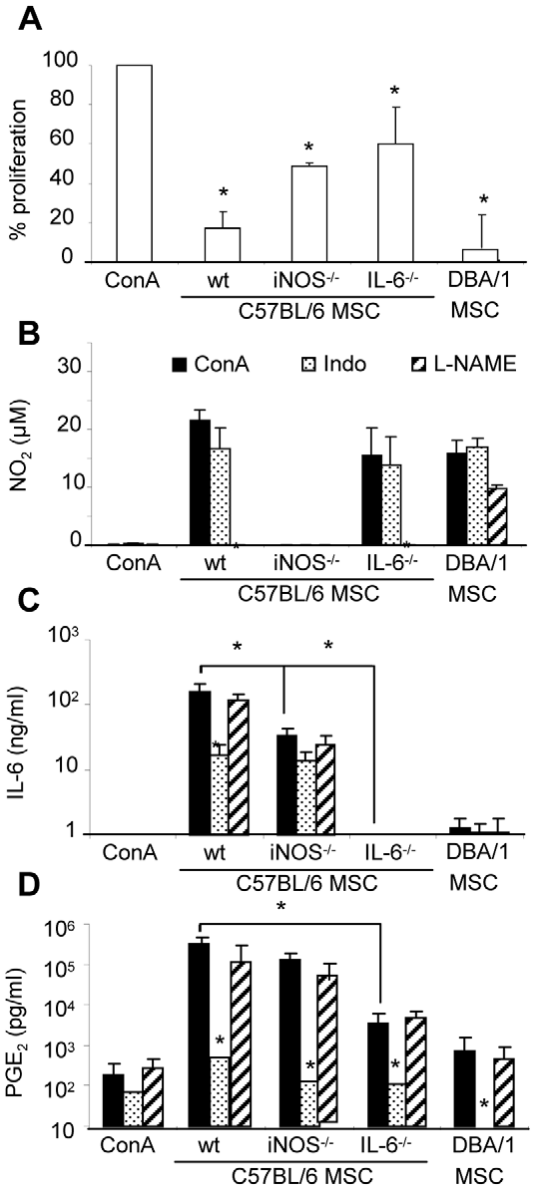
In vitro evaluation of the immunosuppressive potential of MSCs by quantification of soluble mediators. A) Inhibition of T cell proliferation following co-culture with MSCs. Allogeneic splenocytes (10^5^ cells) were incubated for three days with ConA and when indicated, 5×10^4^ wildtype (wt) C57Bl/6 or MSCs isolated from iNOS^−/−^, IL-6^−/−^ knock-out C57Bl/6 mice or DBA/1 MSCs were added. Results are expressed as the percentage of ConA-induced proliferation which was assigned the value of 100% ± SEM (n = 3). B) Quantification of NO_2_ secretion using a modified Griess reagent. C) Quantification of IL-6 secretion by ELISA. D) Quantification of PGE_2_ secretion by ELISA. Levels of the various soluble mediators were quantified in supernatants of the proliferative assays performed in A) after stimulation with ConA. Parallel assays were performed on cells incubated in the same conditions with the addition of inhibitors specific for NO_2_ (L-NAME) or PGE_2_ (indomethacin; Indo). Results are expressed as the mean of 3 independent experiments ± SEM; p values referred to ConA-activated samples (black histograms), if not indicated (*: p<0.05).

Among the mechanisms proposed to mediate the immunosuppressive function of MSCs, IDO, iNOS and COX2, through PGE2 secretion, activities are consistently reported as the most important mediators, at least *in vitro* [21]. IL-6 is another major player as it has been proposed to regulate COX2 function as well as the generation and maturation of dendritic cells [9]–[22] and to be involved in the generation of Treg cells. In order to better evaluate the role of these factors, we derived MSCs isolated from the bone marrow of C57BL6 mice deficient for iNOS or IL-6, respectively named IL-6^−/−^ and iNOS^−/−^, and tested their suppressive potential *in vitro*. IL-6^−/−^ and iNOS^−/−^ MSCs displayed the same phenotypic characteristics and differentiation potential as wild type (wt) B6 MSCs (data not shown). Using a proliferative assay, we found that iNOS^−/−^ and IL-6^−/−^ MSCs were still able to inhibit the proliferation of allogeneic splenocytes, albeit to a significantly lesser extent as compared to wt B6 and D1 MSCs (Fig. 4A). The addition of both iNOS^−/−^ and IL-6^−/−^ MSCs in the proliferative assay did not show any additive effect on the reversion of immunosuppression (data not shown). The immunosuppressive effect was not directly correlated to the secretion of NO as all MSC populations secreted approximately 20 µM NO_2_, except for iNOS^−/−^ cells (Fig. 4B). NO secretion however was only observed when MSCs were cocultured with activated splenocytes. The secretion of NO_2_ was inhibited after addition of the specific inhibitor L-NAME. Splenocytes or MSCs did not express basal levels of IL-6. However, except for IL-6^−/−^ MSCs, IL-6 secretion was induced when MSCs were stimulated by activated splenocytes, whereas it was partly inhibited in presence of the COX2 inhibitor indomethacin (Fig. 4C). Production of IL-6 by iNOS^−/−^ MSCs was 5 fold lower than wt B6 MSCs. Secretion of PGE2 was up-regulated by more than 300 fold when splenocytes were incubated with naïve MSCs, as compared to basal secretion. iNOS^−/−^ and IL-6^−/−^ MSCs, respectively, secreted 2.5 and 80 fold less PGE_2_ than wt B6 MSCs (Fig. 4D). Specificity of the PGE_2_ secretion was confirmed by the neutralization of this production by indomethacin. The production of PGE_2_ was positively correlated with IL-6 secretion and was inversely proportional to the anti-proliferative effect of the MSCs. This is further correlated by the observation that wt D1 MSCs secreted lower levels of IL-6 and PGE2 and were less immunosuppressive at a MSC:splenocyte ratio of 1∶20 (Fig. 1C). Finally, we checked that IDO was not expressed by murine MSCs, both at the mRNA and protein level, as evaluated by RT-qPCR, spectrophotometric assay and high performance liquid chromatography (data not shown). These results suggest that whereas NO is partly responsible for the anti-proliferative effect of MSCs, IL-6-activated PGE_2_ secretion mainly participates in this function.

### iNOS^−/−^ and IL-6^−/−^ MSCs partially suppress arthritis-associated inflammation

**Figure 5:**
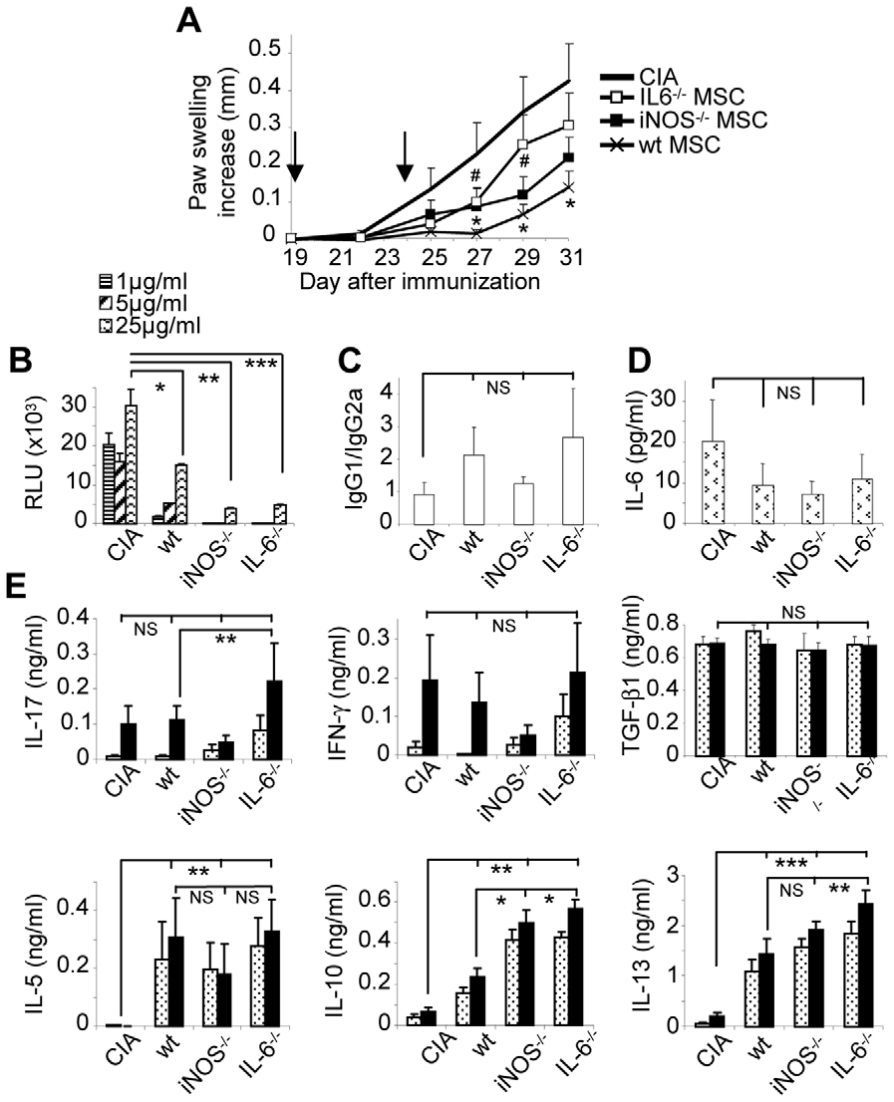
Immunosuppressive effect of iNOS^−/−^ and IL-6^−/−^ MSCs in experimental arthritis. A) Evaluation of the severity of arthritis by measurement of the increase of the paw swelling of control mice (CIA) or mice injected with wt MSCs, iNOS^−/−^ or IL-6^−/−^ MSCs at d18 and d24 (see arrows). Results are expressed as mean increase ± SEM and representative of three independent experiments (*: p<0.05 between wt MSC-treated group and CIA group; #: p≤0.05 between wt MSC-treated group and IL-6^−/−^ MSC-treated group). B) T-cell proliferation assay using splenocytes (10^5^ cells) from the various treated mice incubated with 1, 5 or 25 µg/ml of bCII. C) Determination of the ratio of the bCII-specific IgG1/IgG2a by ELISA in mouse sera. Results are expressed as mean ± SEM. D) Quantification of IL-6 by ELISA in the mouse sera. E) Quantification of cytokines secreted by 2×10^6^ splenocytes cultured *in vitro* without activation (dashed histograms) or stimulated with 10 µg/ml bCII (black histograms) for 24 h (IFN-γ and IL-17) or 48 h (others) by ELISA. Results are expressed as the mean ± SEM (*: p<0.05; **: p<0.01; ***: p<0.005; NS: not significant).

We then compared *in vivo* the injection of iNOS^−/−^ or IL-6^−/−^ MSCs to that of wt B6 MSCs in the allogeneic CIA model. Both deficient cell populations were less efficacious than wt MSCs in decreasing the paw swelling of the arthritic mice and wt MSCs were the only cell population able to significantly reduce the paw swelling of treated mice (Fig. 5A). However, IL-6^−/−^ MSCs, but not iNOS^−/−^ MSCs, were able to induce a significant reversal of immunosuppression as compared to wt MSCs. We therefore investigated whether MSCs were able to inhibit the T cell proliferation and to differentially regulate the involvement of Th1 or Th2 lymphocytes. Irrespective of the nature of the injected MSCs, the bCII-specific response was decreased as assessed by the significantly lower bCII-specific proliferative responses of cells isolated from spleen (Fig. 5B) and draining lymph nodes (DLN) (data not shown). Concomitantly, the bCII-specific IgG1/IgG2a ratio tended to be higher in MSC-treated mice sera than in CIA control sera with no significant differences between groups of MSC-treated mice (Fig. 5C). Serum IL-6, reflecting systemic inflammation, also showed the tendency to decrease in all groups of MSC-treated mice (Fig. 5D).

As naïve CD4^+^ T cells can differentiate into Th1 or Th2 effector cells displaying a distinct cytokine profile, cytokine production levels were thus quantified in supernatants from non activated or bCII-stimulated cells isolated from spleen and DLN. The production levels of the pro-inflammatory cytokines IL-17 and IFN-γ secreted by splenocytes were unchanged in MSC-treated mice as compared to CIA control mice, except for a significantly higher level of IL-17 in IL-6^−/−^ MSC-treated mice (Fig. 5E). In contrast, a high secretion of the anti-inflammatory cytokines IL-5, IL-10 and IL-13 was detected in spleen of MSC-treated mice as compared to CIA control group, whereas TGF-β1 secretion was not affected (Fig. 5E). Secretion of IL-10 and IL-13 was significantly higher in IL-6^−/−^-treated group than in wt MSC-treated group, whereas IL-13 production was only increased in IL-6^−/−^-treated mice. Locally, in the DLN, decreased IL-17, IFN-γ and increased IL-5, IL-10, IL-13 secretion was also observed (data not shown). Taken together, all the immunological parameters tested suggest that MSCs induced a switch of the immune response towards a Th2 cytokine production profile both at the local and systemic level.

### The immunosuppressive effect of MSCs is independent on Treg cell induction

**Figure 6:**
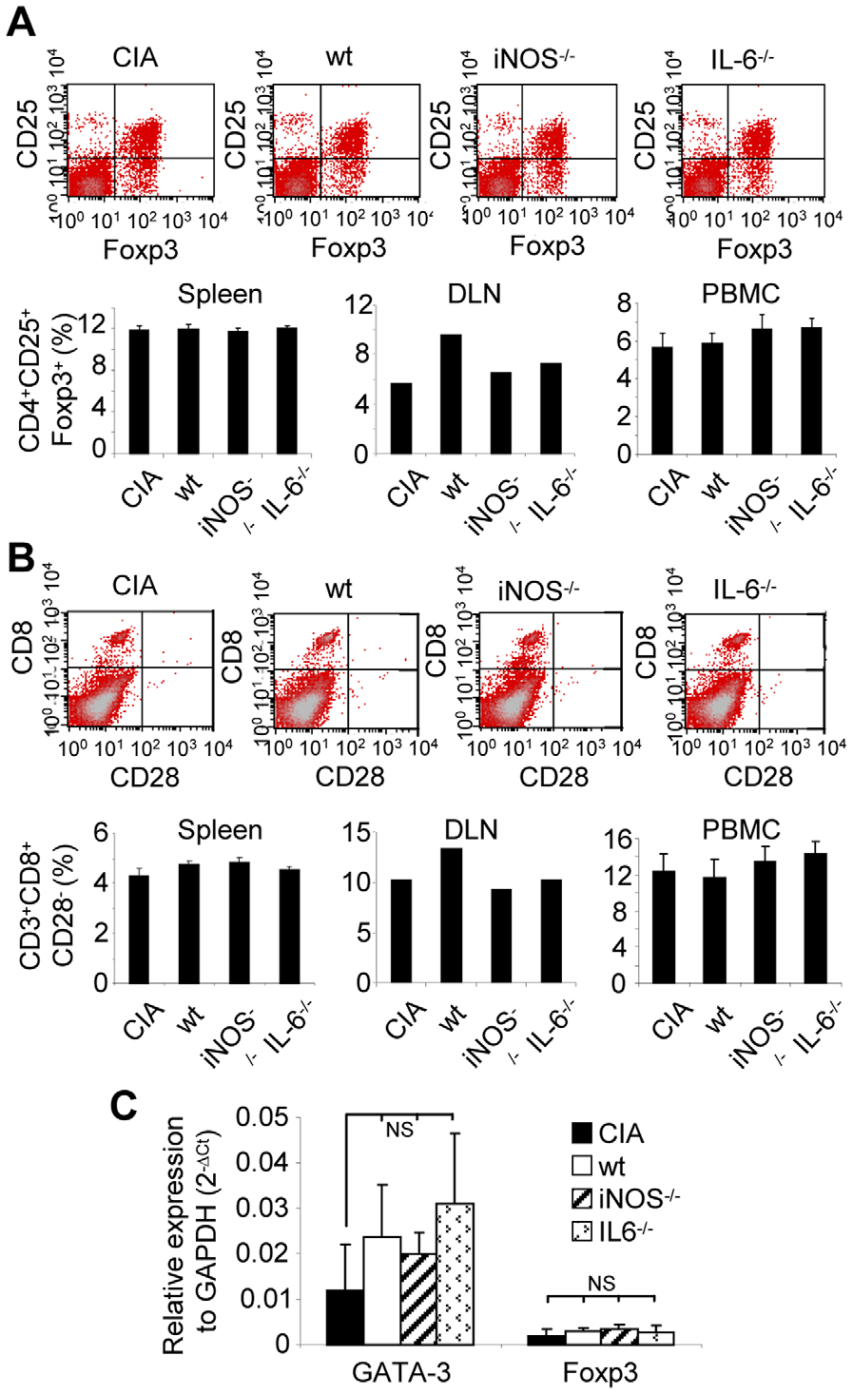
Induction of a Th2 cellular response after treatment with MSCs. A) Percentage of CD4^+^CD25^+^Foxp3^+^ Treg cells in individual spleen, pooled draining lymph nodes (DLN) and individual peripheral blood mononuclear cells (PBMC) from control CIA or MSC-treated mice at day 31. FACS analyses show CD25^+^Foxp3^+^ cells gated on CD4^+^ T cells in the splenocyte population. B) Percentage of CD3^+^CD8^+^CD28^−^ Treg cells in spleen, DLN and PBMC from control CIA or treated mice at day 31. For DLN, cells isolated from all mice in each treated group were pooled resulting in absence of SEM. Results are shown as representative dot plots for 1 mouse per group of mice (upper panel) and histograms in lower panel (% ± SEM), representative of three independent experiments of FACS analyses. C) Relative expression of mRNA level of GATA-3 and Foxp3 transcription factors to GAPDH mRNA in splenocytes using the formula 2^−ΔCt^. Results are expressed as the mean ± SEM (NS: not significant).

To verify whether the suppressive effect of MSCs was exclusively dependent on the induction of Th2 lymphocytes, we looked for the induction of regulatory cells in spleen and DLN of injected mice. Classical Treg cells are defined as CD4^+^CD25^+^Foxp3^+^ cells although the induction of CD8^+^CD28^−^ or CD8^+^CD28^+^ Treg cells has also been shown to be induced by MSCs [23]. Approximately 12% of cells from spleen and 6% from DLN and PBMC from mice injected or not with MSCs were found to be CD4^+^CD25^+^Foxp3^+^ Treg cells. This percentage was not modified after the administration of MSCs irrespective of their origin (Fig. 6A). Similarly, the percentages of CD8^+^CD28^−^ cells (Fig. 6B) or CD8^+^CD28^+^ cells (approximately 3% in spleen and 0.2% in DLN) were not affected by MSC injection. Because subsets of B lymphocytes or macrophages characterized by IL-10 secretion have been attributed a regulatory function, we investigated whether such cell subsets were induced by MSCs. The proportion of IL-10-producing F4-80^+^ macrophages and B220^+^ B lymphocytes was approximately 1% and 50% respectively, among cells from spleen and did not vary following MSC treatment. Finally, the induction of T cell subsets is associated with the up-regulation of subset-specific transcription factors. The expression of transcripts for Foxp3 by Treg cells and GATA-3 by the Th2 subset was thus assessed in splenocytes of the various groups of mice by RT-qPCR. The expression level of GATA-3 tended to be higher in MSC-treated mice, independently of the lack of expression of IL-6 or NO, as compared to the CIA control, while Foxp3 levels remained unchanged between groups (Fig. 6C). Altogether, the data confirmed that MSCs mediated a therapeutic effect independently of the induction of Treg cell subset and was associated with a polarization of the host immune response into a Th2 profile, independently of IL-6 or NO secretion.

## Discussion

The immunosuppressive effect of MSCs is of great interest for the prevention of transplant rejection or graft versus host disease [13], [24]. Successful treatment has also been reported in mice with experimental autoimmune diseases, such as CIA or EAE, the animal models of human RA and multiple sclerosis, respectively [14], [18]. However, conflicting results on the mechanisms developed by MSCs to suppress inflammation are still under debate. In this study, we relied on the use of MSCs isolated from naïve mice and mice deficient for immunosuppressive molecules to investigate the role of these factors *in vitro* and *in vivo*. The main results are i) the differential suppressive effect between MSCs isolated from different mouse strains is only partly dependent on NO and may be mainly attributed to IL-6-activated pathways through PGE2 up-regulation, ii) MSCs display a local intra-articular suppressive effect, in particular through the secretion of IL6-dependent PGE2 and iii) MSCs exert a systemic suppressive effect by mediating a polarization of the host immune response towards a Th2 profile, independently from Treg cell induction or NO and IL-6 secretion.

One important finding of our study is that efficient therapeutic treatment of CIA relies on the injection of MSCs during a narrow window of application (day 18 and 24) as injection of MSCs after disease onset did not prevent the occurrence of arthritic signs. Conflicting results on the therapeutic effect of MSCs have been reported in the CIA model. We and others previously reported that MSC injection failed to reduce arthritis severity [16], [17], [25], [26], in contrast to the results from two other studies that showed a therapeutic benefit [18], [20]. The discrepancy between these studies may be related to the source of cells (cell lines versus primary cells) or various mouse strains, as well as to the dose, route and time of administration. A lack of efficacy of MSC treatment related to the timing of administration has already been reported in a model of GVHD [27]. The authors concluded that factors required for the induction of the immunosuppressive activity of MSCs were not present at the time of injection and that MSC activation was notably dependent on the magnitude of IFN-γ exposure. This is also supported by *in vitro* results indicating that MSCs may behave as antigen presenting cells in presence of low doses of IFN-γ and thus, rather exacerbate the immune response [28]. In RA physiopathogenesis, TNF-α is the major player in disease onset and in the CIA model, TNF-α secretion increases at day 20 to peak at day 30 [29]. We have previously suggested that TNF-α may inhibit the function of MSCs when the cells were injected on day 21 [16]. Accordingly, it has been shown that TNF-α primarily targets mesenchymal cells resulting in the development of chronic inflammatory polyarthritis and the conditional expression of TNFRI in these cells is sufficient to induce a fully arthritic phenotype [30], [31]. These studies therefore suggest that injected MSCs may be activated by TNF-α and play a role in the pathogenesis of arthritis. In the present study, the injection of MSCs on day 18 is likely to prevent immune cell activation which occurs at boost and in particular, to reduce the secretion of TNF-α. As a result, the activation of endogenous or exogenous mesenchymal cells by TNF-α will not take place, resulting in the inhibition of arthritis development. Immunosuppression requires the presence of MSCs for a time lag sufficient for immune cell education. Indeed, migration of MSCs to target tissues and secondary lymphoid organs where they are able to educate immune cells is likely to be required for efficient therapy. This has not been tested here but would require further investigation as the role of chemokines has been reported to be necessary to the immunosuppressive effect of MSCs in a model of GVHD [5]. Finally, a recent study demonstrated that MSCs can be polarized into two acting phenotypes classified as MSC1 and MSC2. TLR4-primed MSCs, or MSC1, mostly elaborate pro-inflammatory mediators, while TLR3-primed MSCs, or MSC2, are mostly immunosuppressive [32]. This study provides a possible explanation to some of the conflicting reports on the immune modulating properties of MSCs.

In the present study, the therapeutic effect was independent from MHC haplotype since we obtained similar results using syngeneic or allogeneic MSCs. However, previous studies relying on the use of primary MSCs obtained using similar protocols as the one used here, although in different animal models, have shown that allogeneic MSCs may lose their suppressive effect *in vivo* and, actually, will elicit an immune response from the host [33], [34]. To better correlate *in vitro* and *in vivo* data, we decided to rely on fully characterized BM-derived primary MSCs which led us to show here that MSCs obtained from different strains of mice do not exhibit the same capacity to inhibit T cell proliferation *in vitro*. D1 MSCs were less efficient in inhibiting T cell proliferation than B6 MSCs. This was shown in proliferative assays using various ratios of splenocytes versus MSCs and was confirmed using quantitative assays for specific immunosuppressive mediators. This effect was proportional to the levels of IL-6 and PGE2 secreted by the various types of MSCs. Thus, D1 and IL-6^−/−^ MSCs produced the lowest levels of PGE2 and IL-6. The secretion of PGE2 was enhanced proportionally to the expression levels of IL-6 in both wt and iNOS^−/−^ MSCs. Conversely, PGE2 inhibition by indomethacin partly inhibited IL-6 secretion by MSCs. This feedback regulatory loop has already been reported in other model systems [35]. The key role of PGE2 in MSC-mediated immunosuppression is supported by results from a recent study showing that PGE2 represents the key inhibitory mediator of DC differentiation and maturation and as a consequence, of reduced T cell activation [36]. Moreover, MSC-derived PGE2 was reported to act on macrophages, increasing their IL-10 secretion and reducing inflammation [10]. We have observed that MSCs promote *in vitro* the secretion of IL-10 by macrophages. We also showed that in vitro, iNOS^−/−^ MSCs were less suppressive than wt MSCs. However, these cells secreted lower levels of IL-6 and PGE2 as compared to wt MSCs, making it difficult to appreciate the role of individual molecules. This finding was consistent with recent results showing inhibition of IL-6 and PGE2 after inhibition of NO in cartilage [37]. IL-6 seems to be the central player since it is induced by PGE2 [38] but IL-6 also positively regulates both COX2 and iNOS activities [22], [39]. Indeed, in absence of IL-6 both NO and PGE2 production are reduced. In our model *in vitro*, NO secretion was however poorly impaired by loss of IL-6. Therefore *in vitro*, we showed that iNOS^−/−^ MSCs exert a lower suppressive activity than wt MSCs. Using IL-6^−/−^ MSCs, we demonstrated that IL-6-dependent PGE2 secretion is correlated with the immunosuppressive activity of MSCs suggesting that PGE2 is the major mediator of immunosuppression.

In the CIA model, we confirmed the role of IL-6 and PGE2 in the suppressive activity of MSCs since IL-6^−/−^ MSCs exhibited the lowest suppressive effect and did not significantly reduce the severity of arthritis as compared to wt MSCs. Decreased IL-6-dependent PGE2 secretion could account for this effect as observed *in vitro* through its role on T cell proliferation, macrophage reprogramming or DC maturation [40], [41], [42]. Here, the effect of MSC-mediated iNOS activity was marginal since iNOS^−/−^ MSCs behave similarly as wt MSCs. This result differs from another study in the experimental model of GVHD [5]. The authors however principally used MSC clones which behave differently from populations since the clones did not secrete PGE2. It could be hypothesized that the inability of iNOS^−/−^ MSCs to suppress immune responses may be the result of their impaired function to secrete both NO and PGE2 which may be mediators with complementary immunosuppressive functions. Therefore, using for the first time IL-6-deficient MSCs, we confirmed *in vivo* that MSC immunosuppression was mainly dependent on IL-6-activated signalling pathways although this effect was partial and that, PGE2 is likely to be the mediator acting downstream in the MSC-mediated immunomodulation.

Surprisingly, the clinical differences observed using various MSC types were not concordant with the biological responses since the evaluation of local and systemic immune responses of MSC-treated mice revealed similar profiles, irrespective of their production of either NO or IL-6. We observed a decreased bCII-specific T cell proliferative response, an increased bCII-specific IgG1/IgG2a ratio and low levels of pro-inflammatory mediators, as well as increased levels of anti-inflammatory cytokines in bCII-primed T lymphocytes from spleen. All these parameters are characteristic of a Th2 immune profile which was further confirmed by the increase in GATA-3 mRNA levels in splenocytes from MSC-treated animals. These data were not consistent with those of a previous study indicating that secretion of IL-10 and IL-4 was down-regulated [18]. In the latter study however, MSCs were cultured *in vitro* with T lymphocytes isolated from immunized mice and challenged with bCII for 2 days. Because a Treg cell-dependent mechanism of suppression has been proposed in CIA [18], [20], [43], we investigated the increase of CD4^+^CD25^+^Foxp3^+^, CD3^+^CD8^+^CD28^+^ or CD3^+^CD8^+^CD28^−^ cells in spleen, DLN and blood. No significant increase of these T cell populations was observed and no changes in TGF-β1 secretion levels or in Foxp3 mRNA expression were observed in splenocytes, further suggesting that no Treg cells were induced. The discrepancy between previous results and those reported here may arise from the time and route (intra-peritoneal versus intravenous) of MSC administration leading to variable immune responses. Interestingly, injection of CD4^+^CD25^+^ Treg cells did not alter bCII-specific antibody secretion or T cell proliferative response while markedly slowing CIA progression [44]. Our results demonstrating altered bCII-specific T and B cell responses are not in line with the hypothesis of Treg cell induction but are in concordance with this last study. Lack of Treg cell induction by MSCs was also reported in autoimmune enteropathy [45] and EAE [14]. In the latter study, the authors detected a slight down-regulation of CD40 and MHC class II molecules on DCs, suggesting that impaired co-stimulation by DCs from treated mice could contribute to peripheral T cell anergy. Alternatively, secretion of PGE2 by the injected MSCs may impair the maturation of DCs and therefore may account for a switch towards a Th2 immune response profile, as already reported in previous studies [40], [41], [42]. Indeed, anti-proliferative mediators such as NO and more importantly, IL-6-dependent PGE2 may act locally by inhibiting the proliferation of immune cells in the synovium thereby reducing local inflammation. Altogether, our results suggest that NO or PGE2 mediators may act together to decrease local inflammation but that the main effect of MSCs is likely on systemic immunity through a polarization of the host immune response towards a Th2 cytokine production profile.

This study demonstrates that MSCs might be used for regulating inflammatory responses and could offer therapeutic benefit in autoimmune diseases. Like Treg cells, MSCs migrate to the joints where they can act locally inside the inflamed synovium to decrease the proliferation and function of immune cells via the secretion of inhibitory soluble factors. They can also act systemically to suppress the host immune response through a shift in the Th1/Th2 cell balance, indicating that MSC-induced immune suppression is not mediated by a single or unique mechanism. This may have important therapeutic applications far beyond the field of autoimmune diseases.

## Materials and Methods

### Isolation of stromal cells

MSCs from C57BL/6 or DBA/1 mice or from iNOS- or IL-6-knock-out C57BL/6 mice were isolated from bone marrow (BM). BM was flushed out from long bones and the cell suspension (0.5×10^6^cells/cm^2^) was plated in minimum essential medium (MEM)-α supplemented with 10% fetal bovine serum (FBS) (Hyclone, Thermo Fisher Scientific, Brebières, France), 2 mM glutamine, 100 U/mL penicillin, 100 mg/mL streptomycin (Lonza, Levallois-Perret, France) and 2 ng/ml human basic fibroblast growth factor (bFGF) (R&D Systems, Lille, France). At sub-confluence, cells were collected, propagated at a density of 5,000 cells/cm^2^ and used between passages 6 and 10.

### Differentiation of MSCs

Differentiation of MSCs was induced by culture under specific conditions for 21 days. For adipogenesis, MSCs were plated at 10^4^ cells/cm^2^ in complete Dulbecco's modified Eagle's medium (DMEM)-F12 (Invitrogen) with 16 µM biotin, 18 µM panthotenic acid, 100 µM ascorbic acid, 5 µg/ml insulin, 0.03 µM dexamethasone, 1 µg/ml transferring, 2 ng/ml triiodothyronine (T3) and 100 nM rosiglitazone (Sigma-Aldrich, Saint-Quentin Fallavier, France). Formation of lipid droplets was visualized by Oil red O staining on cells fixed by 3% glutaraldehyde for 1 h. For osteogenesis and chondrogenesis, inductive conditions were already reported [46]. Chondrogenesis was assessed by RT-qPCR and immunohistochemistry on paraffin sections of pellets using a 1/50 dilution of anti-aggrecan antibody (Chemicon, Millipore, Molsheim) and the "Ultravision detection system anti-polyvalent HRP/DAB" kit (Lab Vision, Francheville, France). Sections were counterstained with Mayer's hematoxylin (Lab Vision) for 3 min and mounted with Eukitt (Sigma-Aldrich). Osteogenic differentiation of MSCs was assessed by RT-qPCR and extracellular matrix mineralization detected as already described [46].

### RT-qPCR analysis

Total RNA was extracted using the RNeasy mini kit (Qiagen S.A., Courtaboeuf, France). RNA (500 ng) was reverse transcribed using the Multiscribe reverse transcriptase and PCR was done with the GeneAmp® RNA PCR Core Kit using the "Assays-on-Demand" gene expression assays (Applied Biosystems, Courtaboeuf, France) on the Lightcycler 480 (Roche Applied Systems, Meylan). Content of cDNA samples was normalized to the expression of GAPDH mRNA and expressed either as relative expression to GAPDH mRNA using the formulae 2^−ΔCt^ or as fold increase using the formula 2^−ΔΔCt^.

### Flow Cytometry analysis

MSCs (5×10^5^ cells) were suspended in phosphate-buffered saline (PBS) containing 0.1% bovine serum albumin and 0.01% sodium azide and incubated for 20 min on ice with conjugated monoclonal antibodies. Specific and isotypic control antibodies were from BD Biosciences (Le Pont de Claix, France).

Mononuclear cell suspensions were isolated after dissociation of spleens and draining lymph nodes or from blood after Ficoll separation as described elsewhere [47]. For membrane staining, cells were incubated with anti-mouse conjugated antibodies or F4-80 or B220 conjugated antibodies (BD Biosciences) as described above. For Foxp3 intracellular staining, cells were incubated in 150 µl/well of Fix/Perm solution overnight at 4°C and then at 4°C for 15 min with permeabilization buffer (Cliniscience, Montrouge, France), followed by incubation with anti-Foxp3 antibody for 30 min on ice (Miltenyi Biotec, Paris, France). Flow cytometry was performed on a fluorescence activated cell sorter (FACSCalibur), and data analysed with the CellquestPro software (BD, Le Pont de Claix, France).

### Measurement of IDO and iNOS Activities

IDO enzyme activity was measured after MSC stimulation with 1000 U/ml IFN-γ for 48 hours as reported [46], [48].

Because nitric oxide (NO) is quickly converted to NO_2_ and NO_3_ in culture medium, NO_2_ production was measured using a modified Griess reagent (Sigma-Aldrich) as described previously [49].

### Quantification of cytokines

Enzyme-linked immunosorbent assays (ELISA) were from R&D Systems or Cliniscience for IL-6. Cytokines and PGE2 were quantified in culture supernatants from T-cell proliferation assays or in sera. For analysis of cytokine production by splenocytes or lymph node cells, 2×10^6^ cells were seeded in proliferation medium and stimulated with 10 µg/ml of bCII. Supernatants were collected either after 24 h for IFN-γ and IL-17 or 48 h for the others. bCII-specific immunoglobulins were quantified using alkaline phosphatase-labelled anti-mouse IgG1 and anti-mouse IgG2a antibodies (BD Biosciences) as previously described [50].

### T-cell proliferation assay

Splenocytes were obtained after spleen dissociation and lysis of erythrocytes with an equal volume of ACT solution (155 mM NH_4_Cl, 0.1 mM EDTA and 10 mM KHCO_3_). Lymph nodes were disaggregated by treatment with 0.1% collagenase D (Roche) at 37°C for 1 hour. Isolated cells were seeded in triplicates in 100 µl of medium consisted of heat-inactivated FBS-containing RPMI 1640 supplemented with 0.1 mM non essential amino acids, 1 mM sodium pyruvate, 20 mM N-2-hydroxyethylpiperazine-N'-2-ethanesulfonic acid (HEPES) (Invitrogen) and 50 µM 2-mercaptoethanol (Lonza).

For coculture experiments, 10^5^ splenocytes were stimulated with 1 µg/ml concanavalin A (conA) (Sigma-Aldrich). When necessary, 5×10^4^ MSCs per well (ratio 1/2 of MSC/T cells if not indicated or 5×10^3^ MSCs corresponding to ratio 1/20) and/or inhibitors (indomethacine 5 µM; L-NAME 10 mM, Sigma-Aldrich) were added. For evaluating the proliferation of T cells isolated from *in vivo* experiments, 8×10^5^ splenocytes or cells from lymph nodes were stimulated by 5 µg/ml of conA or 1, 5, 25 µg/ml of bCII.

After 3 days, cell proliferation was measured using the CellTiter-Glo™ luminescent cell viability assay (Promega, Charbonnières-les-Bains, France).

### Arthritis induction and measurement

Adult male DBA/1 mice aged 9–10 weeks were grown in our animal facilities. All animal experiments complied with the regulations of the Ethical Committee of the Languedoc-Roussillon. Approval CEEA-LR-1042 (Comité Régional d'éthique pour l'expérimentation animale, Languedoc Roussillon). Immunization was performed as reported in [16] and MSCs (1×10^6^ cells) were injected intravenously on day 18 and 24, otherwise indicated. Signs of arthritis were assessed by measuring the paw swelling of the hind paw and evaluating the clinical score using the macroscopic scale as previously described [16]. After sacrifice, the hind limbs were collected for X-ray and radiological scoring was performed as described [51].

### Histology and immunohistochemistry

The paws were fixed in 4% paraformaldehyde, decalcified overnight in Rapid Bone Decalcifier (Eurobio, Les Ulis, France) and processed for routine histology. Histological scoring was performed on hematoxylin/eosin/safranin O sections as follows: 0, normal; 1, inflammatory infiltrates and synovial hyperplasia; 2, pannus formation and cartilage erosion; 3, important cartilage erosion and bone destruction, 4, loss of joint integrity.

### Statistical analysis

Statistics were done using the Student t test and for *in vivo* experiments, with an impaired Mann-Whitney test to compare nonparametric data for statistical significance.
